# Preoperative predictors for recurrence sites associated with poor post-recurrence survival after surgery of non-small cell lung cancer: a multicenter study

**DOI:** 10.1186/s12885-023-11582-y

**Published:** 2023-11-06

**Authors:** Tetsuya Isaka, Hiroyuki Adachi, Kotaro Murakami, Jun Miura, Noritake Kikunishi, Naoko Shigeta, Yujin Kudo, Yoshihiro Miyata, Morihito Okada, Norihiko Ikeda, Hiroyuki Ito

**Affiliations:** 1 Department of Thoracic Surgery, Kanagawa Cancer Center, 2-3-2 Nakao, Asahi, Yokohama, Kanagawa 241-8515 Japan; 2 Department of Surgery, Tokyo Medical University, Tokyo, Japan; 3 Department of Surgical Oncology, Hiroshima University, Hiroshima, Japan

**Keywords:** Initial site of recurrence, Post-recurrence survival, Non-small cell lung cancer, PET maxSUV, CEA

## Abstract

**Background:**

The recurrence site that influences post-recurrence survival (PRS) in patients with non-small cell lung cancer (NSCLC) undergoing surgery and the preoperative predictors of recurrence remain unclear.

**Methods:**

Cohorts 1 and 2 had 4520 (who underwent complete resection for p-stage 0-IIIA NSCLC) and 727 (who experienced recurrence after surgery) patients, respectively. The initial sites of recurrence were the lungs (309 cases), thoracic lymph nodes (225 cases), pleura (112 cases), bone (110 cases), central nervous system (86 cases), adrenal gland (25 cases), abdomen (60 cases), cervical and axillary lymph nodes (38 cases), chest wall (13 cases), skin (5 cases), and eye and tongue (3 cases). For cohort 2 analysis, the initial recurrence site that resulted in poor PRS was analyzed by multivariable analysis using a Cox proportional hazard model. For cohort 1 analysis, the preoperative predictors of recurrence patterns with poor PRS were analyzed by multivariable analysis using a logistic regression model.

**Results:**

In cohort 2 analysis, recurrence in the central nervous system (hazard ratio [HR], 1.70; *p *< 0.001), bone (HR, 1.75; *p* < 0.001), abdomen (HR, 2.39; *p* < 0.001), and pleura (HR, 1.69; *p* < 0.001) were independent poor prognostic recurrent sites for PRS and they were high-risk sites (HRS). Intrathoracic lymph nodes, cervical and axillary lymph nodes, lungs, chest wall, adrenal gland, eye and tongue, and skin were low-risk sites (LRS) that did not affect PRS. Patients with multiple LRS without HRS recurrence had a worse prognosis than those with a single LRS without HRS recurrence (5-year PRS 20.2% vs. 37.7%, *p* < 0.001) and were comparable to those with HRS recurrence (*p* = 1.000). In cohort 1 analysis, preoperative predictors for HRS and multiple LRS recurrences were positron emission tomography (PET) maximum standardized uptake value (maxSUV) ≥ 3.2 (HR, 5.09; *p* < 0.001), clinical nodal metastasis (HR, 2.00; *p* < 0.001), tumor size ≥ 2.4 cm (HR, 1.96; *p* < 0.001) and carcinoembryonic antigen (CEA) ≥ 5 ng/ml (HR, 1.41; *p* = 0.004). The cumulative incidence rates of HRS and multiple LRS recurrences within 5 years were 55.9%, 40.9%, 26.3%, 11.1%, and 3.5% (*p* < 0.001) in patients with 4, 3, 2, 1 and 0 of the above risks, respectively.

**Conclusions:**

HRS and multiple LRS were vital recurrences associated with poor PRS. Preoperative PET maxSUV, clinical nodal metastasis, tumor size, and CEA level predicted the incidence of vital recurrence.

**Supplementary Information:**

The online version contains supplementary material available at 10.1186/s12885-023-11582-y.

## Background

Lung cancer is the leading cause of death worldwide [1]. Among patients with non-small cell lung cancer (NSCLC) who undergo surgery, 30–55% show recurrence [2, 3]. The standard treatment for patients with NSCLC recurrence is similar to that for advanced-stage lung cancer, with a poor post-recurrence survival (PRS); median PRS of 17.6–30 months [3–6], and a 5-year PRS of 18.8–31.9% [3–7]. The local and distant recurrences have been reported in approximately 24–38% and 40–78% of cases, respectively [3, 4, 6].

Male sex, older age, smoking history, low-performance status, short-term recurrence, histological presence of symptoms, and poor differentiation have been reported to be poor prognostic factors for PRS [3–6, 8]. However, there is still no consensus regarding the relationship between PRS and the site of recurrence. There is also no consensus on the risk of recurrence at specific sites. Previous studies have reported that extrathoracic or distant recurrence is associated with poor prognosis [9, 10]. Other studies have reported that patients with local recurrence had a survival advantage over those with distant recurrence [7, 8, 11]; however, in some studies, distant recurrence did not affect PRS [6, 12, 13].

Analyzing recurrence sites that result in poor prognosis is necessary for personalized postoperative surveillance. Moreover, predicting these vital recurrences is important in selecting patients for neoadjuvant/adjuvant therapy. In recent years, several clinical trials for preoperative treatment of clinical stage IB-III NSCLC have been conducted, including CheckMate-816, AEGEAN, CheckMate-77T, KEYNOTE-671, IMpower030, and NeoADAURA, and evidence is gradually being established [14]. Preoperative prediction of vital recurrence is important in selecting patients who should receive neoadjuvant or adjuvant therapy.

This study aimed to identify vital recurrence resulting in poor PRS in patients with postoperative recurrence of NSCLC using a multicenter database and to analyze the preoperative predictors of vital recurrence.

## Methods

### Ethics statement

The study adhered to the tenets of the Declaration of Helsinki. The institutional review boards of the participating institutions approved this retrospective review of a prospective database and waived the requirement for informed consent for each patient (Kanagawa Cancer Center, approval 24EKI54; Tokyo Medical University Hospital, approval SH2969; Hiroshima University Hospital, approval E-1216).

### Patients and study design

**Fig. 1:**
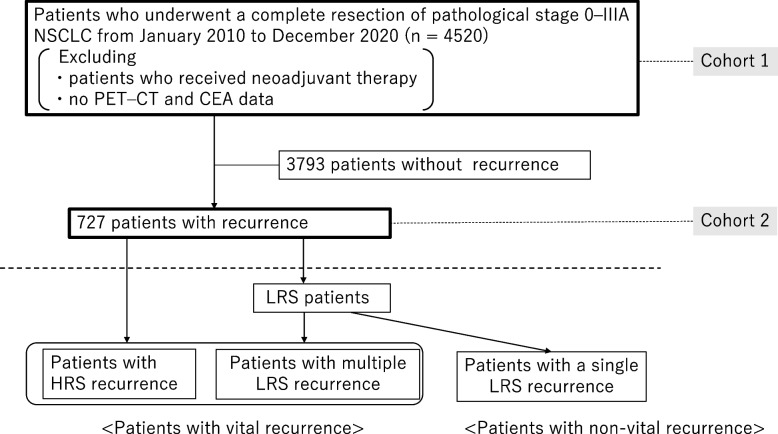
Consort diagram of this study. *HRS* High-risk site, *LRS* Low-risk site, *NSCLC* Non-small cell lung cancer

In this study, 4520 patients who underwent complete resection for pathological stage 0-IIIA NSCLC at Kanagawa Cancer Center, Hiroshima University Hospital, and Tokyo Medical University between January 2010 and December 2020 were included in cohort 1 (Fig. 1). This study excluded patients who received neoadjuvant therapy and who have unavailable information on positron emission tomography (PET) maximum standardized uptake value (maxSUV) or carcinoembryonic antigen (CEA) level (Fig. 1). Among the 4520 patients, 727 experienced recurrences after surgery and were included in cohort 2 (Fig. 1). The present study had the following two parts: First, the recurrence patterns that resulted in poor PRS, namely vital recurrence, were examined in cohort 2. Second, the preoperative predictors of vital recurrence were analyzed in cohort 1.

### Categorization of the initial recurrence sites and word definitions

The initial recurrence site was defined as recurrent organs that could be identified by diagnostic imaging performed before treatment for recurrence. The initial metastatic organs were classified into the following ten recurrence site categories: (1) thoracic lymph node recurrence, including mediastinal and hilar lymph node recurrence; (2) cervical and axillary lymph node recurrence, including sub/supraclavicular lymph node; (3) lung recurrence, including ipsilateral or contralateral lung recurrence; (4) pleural recurrence, including pleural dissemination; (5) chest wall recurrence; (6) bone recurrence; (7) central nervous system (CNS) recurrence, including brain metastasis and meningeal dissemination; (8) adrenal recurrence; (9) abdominal organ recurrence, including liver, pancreas, intestine, and intraperitoneal lymph node recurrence; (10) skin recurrence; and (11) eye and tongue recurrence.

PRS was defined as the duration from the first evidence of relapse to the time of all-cause death, censoring patients without an event during the last observation period. Overall survival (OS) was defined as the period from the date of surgery to the date of all-cause death, wherein patients were censored without any events in the last observation period.

### Statistical analysis

Continuous variables were compared using the Mann–Whitney U test and categorical variables were compared using Fisher’s exact test. OS and PRS were analyzed using the Kaplan–Meier method and compared between the groups using log-rank tests. Cut-off values for the computed tomography (CT) tumor size, Brinkmann index, and PET maxSUV were determined using receiver operating characteristic curve analysis in cohort 1.

In cohort 2, univariable and multivariable analyses of PRS were performed using the Cox proportional hazard model to analyze the following variables: age (≥ 65 years), sex, histology, smoking history, CT tumor size, surgical procedure, pathological stage, lymphatic invasion, vessel invasion, pleural invasion, nodal metastasis, and each recurrence site. All variables with a *p*-value of < 0.10 in the univariable analysis were analyzed in the multivariable analysis.

In cohort 1, multivariable analysis was performed to examine the predictors of vital recurrence using logistic regression analysis of the preoperative variables: age (≥ 65 years), sex, Brinkmann index, CEA, laterality, tumor location, CT tumor size, PET maxSUV, clinical nodal metastasis, and surgical procedure. The cumulative incidence of vital recurrence according to risk was analyzed using Gray’s test.

Statistical significance was set at *p*-value of < 0.05. Statistical analyses were performed using EZR on R commander version 1.30 (Saitama Medical Center, Jichi Medical University, Saitama, Japan), which is a graphical user interface for R (The R Foundation for Statistical Computing, Vienna, Austria).

## Results

The median observation period, median PRS, and 5-year PRS rate of cohort 2 were 32 (19–54) months, 26 months, and 24.5%, respectively. The median age of the patients was 72 years (range, 65–77 years), and 512 (70.4%) patients were males (Supplementary Table). There were 309 (42.5%) patients with lung recurrence, 225 (30.9%) patients with intrathoracic lymph node recurrence, 112 (15.4%) patients with pleural recurrence, 110 (15.1%) patients with bone recurrence, 86 (11.8%) patients with CNS recurrence, 25 (3.4%) patients with adrenal recurrence, 60 (8.3%) patients with abdominal organ recurrence, 38 (5.2%) patients with cervical and axillary lymph nodes metastasis, 13 (1.8%) patients with chest wall recurrence, 5 (0.7%) patients with skin recurrence, and 3 (0.4%) patients with eye and tongue recurrence (Supplementary Table).

In univariable and multivariable analyses of cohort 2, CNS recurrence (hazard ratio [HR], 1.70; 95% confidence interval [CI], 1.25–2.33; *p* < 0.001), bone recurrence (HR, 1.75; 95% CI, 1.31–2.35; *p* < 0.001), abdominal organ recurrence (HR, 2.39; 95% CI, 1.68–3.41; *p* < 0.001), and pleural recurrence (HR, 1.69; 95% CI, 1.25–2.27; *p* < 0.001) were poor prognostic factors for PRS, along with older age (≥ 65 years), smoking history, and non-adenocarcinoma histology (Table 1). These four recurrence sites were defined as high-risk sites (HRS), and the other sites (lung, intrathoracic lymph node, cervical and axillary lymph nodes, adrenal gland, chest wall, skin, and eye and tongue) were defined as low-risk sites (LRS).

**Table 1:** Univariable and multivariable analyses of post-recurrence survival of patients with non-small cell lung cancer

Post-recurrence survival Variable	Univariable analysis			Multivariable analysis		
	HR	95% CI	*p* values	HR	95% CI	*p* values
Age (≧65y)	2.24	1.69–2.97	< 0.001	2.17	1.62–2.92	< 0.001
Male	1.64	1.28–2.11	< 0.001	1.12	0.80–1.56	0.512
Smoking history	1.88	1.44–2.46	< 0.001	1.52	1.06–2.18	0.023
CT tumor size	1.06	1.01–1.12	0.022	1.03	0.97–1.08	0.368
Surgical procedure (sublobar)	1.32	1.00–1.75	0.054	1.25	0.91–1.72	0.161
Non-adenocarcinoma	1.91	1.54–2.38	< 0.001	1.50	1.18–1.90	< 0.001
Pathological stage II ≦	1.02	0.82–1.28	0.832			
Lymphatic invasion ( +)	0.85	0.68–1.05	0.122			
Blood vessel invasion ( +)	1.19	0.93–1.51	0.159			
Pleural invasion ( +)	1.01	0.82–1.25	0.903			
Nodal metastasis ( +)	0.88	0.71–1.09	0.229			
Central nerve system	1.34	1.01–1.80	0.046	1.70	1.25–2.33	< 0.001
Bone	1.46	1.11–1.94	0.007	1.75	1.31–2.35	< 0.001
Abdominal organ	2.49	1.77–3.50	< 0.001	2.39	1.68–3.41	< 0.001
Adrenal gland	1.31	0.75–2.28	0.338			
Pleural	1.27	0.96–1.67	0.091	1.69	1.25–2.27	< 0.001
Eye and tongue	3.33	0.83–13.4	0.090	3.12	0.75–13.0	0.119
Skin	1.56	0.39–6.30	0.531			
Chest wall	2.40	1.13–5.07	0.022	1.69	0.78–3.69	0.184
Lung	0.80	0.64–0.99	0.044	0.99	0.78–1.26	0.929
Intrathoracic lymph node	1.04	0.83–1.30	0.713			
Cervical and axillary lymph node	1.41	0.89–2.24	0.147			

*CEA* Carcinoembryonic antigen, *CI* Confidence interval, *CT* Computed tomography, *HR* Hazard ratio

**Fig. 2:**
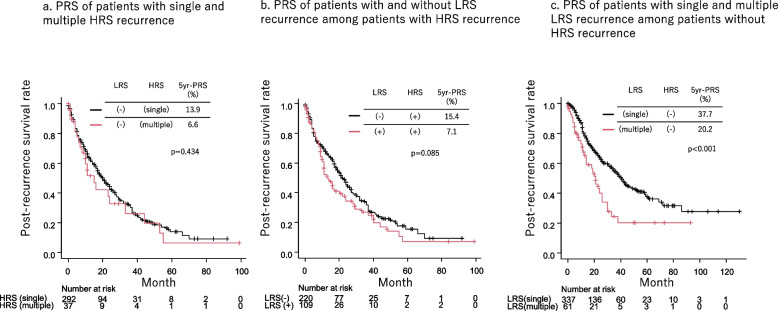
The post-recurrence survival (PRS) of patients with a single high-risk site (HRS) and multiple HRS recurrences were comparable (*p* = 0.434, Fig. 2a). The PRS of patients with both HRS and low-risk site (LRS) recurrence tended to show worse prognosis than that of patient with HRS recurrence without LRS recurrence, but not statistically significant (*p* = 0.085, Fig. 2b). The PRS of multiple LRS recurrent patients were significantly worse than that of single LRS recurrent patients among the patients without HRS recurrence (*p* < 0.001, Fig. 2c)

**Fig. 3:**
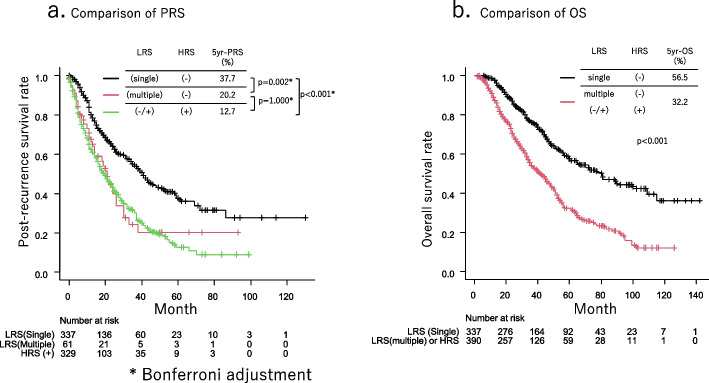
The post-recurrence survival (PRS) of a high-risk site (HRS) recurrent patients was significantly worse than that of a single low-risk site (LRS) recurrent patients (*p* < 0.001, Fig. 3a) and were comparable to that of multiple LRS recurrent patients (*p* = 1.000, Fig. 3a). The overall survival (OS) of patients with HRS and multiple LRS recurrences was significantly worse than that of patients with a single LRS recurrence (*p* < 0.001, Fig. 3b)

The PRS of patients with single and multiple HRS recurrences was comparable (*p* = 0.434, Fig. 2a). The PRS of patients with both HRS and LRS recurrence tended to show worse prognosis than that of patient with HRS recurrence without LRS recurrence, but not statistically significant (*p* = 0.085, Fig. 2b). The PRS of multiple LRS recurrent patients was significantly worse than that of single LRS recurrent patients among patients without HRS recurrence (*p* < 0.001, Fig. 2c). The PRS of patients with HRS recurrence was significantly worse than that of patients with single LRS recurrence (*p* < 0.001, Fig. 3a); however, they were comparable to those of patients with multiple LRS (*p* = 1.000, Fig. 3a).

The characteristics of patients with HRS and multiple LRS recurrences were compared with those of patients with single LRS recurrences (Table 2). Larger tumor size, lymphatic invasion, blood vessel invasion, pleural invasion, and nodal metastasis were more common in the HRS and multiple LRS recurrence groups. Postoperative interval until a relapse was significantly shorter in the HRS and multiple LRS recurrence groups than in the single LRS recurrence group (12-month vs. 17-month, *p* < 0.001). The OS of patients with HRS and multiple LRS recurrences was significantly worse than that of patients with a single LRS recurrence (*p* < 0.001, Fig. 3b).

**Table 2:** Comparison of patients’ clinicopathological characteristics between single low-risk site (LRS) and multiple LRS / high-risk site (HRS)

Total n = 727	Single LRS (*n* = 337)	Multiple LRS / HRS (*n* = 390)	*p* values^a^
Age ≧65y, No. (%)	258 (76.6)	294 (75.4)	0.729
Male, No. (%)	237 (70.3)	275 (70.5)	1.000
Smoking history, No. (%)	247 (73.3)	289 (74.1)	0.866
CT tumor size, median (IQR), cm	3.0 (2.1–4.1)	3.2 (2.4–4.4)	0.038
Non-adenocarcinoma, No. (%)	112 (33.2)	136 (34.9)	0.695
Pathological stage II ≦, No. (%)	203 (60.2)	256 (65.6)	0.143
Lymphatic invasion + , No. (%)	157 (46.6)	219 (56.2)	0.011
Blood vessel invasion + , No. (%)	204 (60.5)	310 (79.5)	< 0.001
Pleural invasion + , No. (%)	156 (46.3)	217 (55.6)	0.014
Nodal metastasis + , No. (%)	145 (43.0)	197 (50.5)	0.045
Postoperative interval until relapse, median (IQR), month	17 (9–31)	12 (7–23)	< 0.001^b^

^a^Fisher’s exact test

^b^Mann-Whitney U test

*CT* Computed tomography, *HRS* High-risk site, *IQR* Interquartile range, *LRS* Low-risk site

**Fig. 4:**
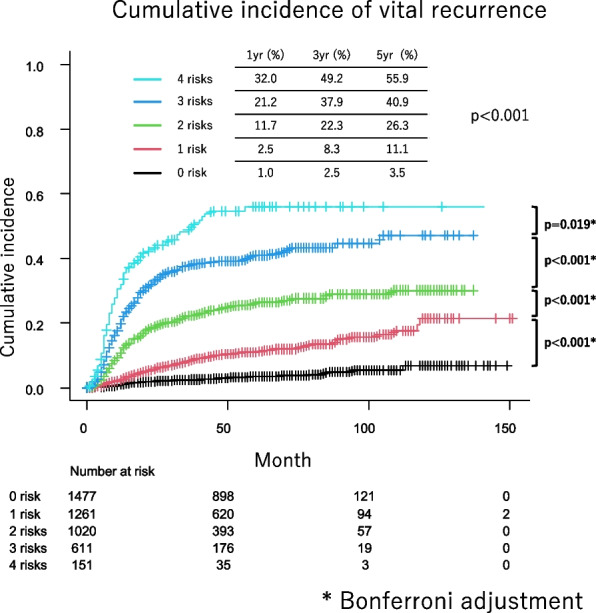
The cumulative incidences of HRS and multiple LRS recurrences at 5 postoperative years were 55.9%, 40.9%, 26.3%, 11.1%, and 3.5% in patients with 4, 3, 2, 1, and 0 of the vital recurrence risks, respectively (*p* < 0.001; Fig. 4)

The median observation period for cohort 1 was 50 (25–67) months. The median patient age was 70 years (range, 64–76 years), and 2550 (56.4%) patients were males (Supplementary Table). In the multivariable analysis in cohort 1, preoperative predictors of HRS or multiple LRS recurrence were CEA ≥ 5 ng/ml (odds ratio [OR], 1.41; 95% CI:1.12–1.77; *p* = 0.004), PET maxSUV ≥ 3.2 (OR, 5.09; 95% CI:3.66–7.08; *p* < 0.001), CT tumor size ≥ 2.4 cm (OR, 1.96; 95% CI:1.50–2.56; *p* < 0.001),and clinical nodal metastasis (OR, 2.00; 95% CI:1.53–2.60; *p* < 0.001) (Table 3). The cumulative incidences of HRS and multiple LRS recurrences at 5 postoperative years were 55.9%, 40.9%, 26.3%, 11.1%, and 3.5% in patients with 4, 3, 2, 1, and 0 of the above risks, respectively (*p* < 0.001; Fig. 4).

**Table 3:** Preoperative predictor of metastasis to high-risk site or multiple low-risk sites

Variable	Multivariable analysis		
	Odd’s ratio	95% CI	*p* values
Age (≧65y)	1.00	0.78–1.30	0.975
Male	1.24	0.94–1.63	0.127
Brinkmann index 600≦	1.03	0.79–1.34	0.822
CEA elevation (5 ng/mL≦)	1.41	1.12–1.77	0.004
Right side	0.89	0.71–1.11	0.308
Tumor location (lower lobe)	1.03	0.82–1.29	0.791
CT tumor size 2.4 cm ≦	1.96	1.50–2.56	< 0.001
PET maxSUV 3.2≦,	5.09	3.66–7.08	< 0.001
Clinical nodal metastasis	2.00	1.53–2.60	< 0.001
Surgical procedure (sublobar)	1.04	0.75–1.44	0.819

*CEA* Carcinoembryonic antigen, *CI* Confidence interval, *CT* Computed tomography, *HR* Hazard ratio, *maxSUV* Maximum standardized uptake value, *PET* Positron emission tomography

## Discussion

This study demonstrated that HRS (CNS, bone, abdominal organ, and pleural) recurrence or multiple LRS (lung, intrathoracic lymph node, cervical and axillary lymph node, adrenal gland, chest wall, eye and tongue, and skin) recurrences were vital recurrences that were associated with poor PRS. Preoperative predictors for vital recurrence were CEA ≥ 5 ng/ml, PET maxSUV ≥ 3.2, CT tumor size ≥ 2.4 cm, and clinical nodal metastasis. Patients with all four predictors had vital recurrence at 55.9% within 5 years after surgery.

The median PRS and 5-year PRS rates were 26 months and 24.5%, respectively, which were similar to those in previous reports [3–6]. In the previous study, the frequency of recurrence to intrathoracic lymph nodes, lung, bone, pleura, brain, adrenal gland, abdominal organ, cervical lymph node, and chest wall was 22–42% [4, 6], 37–42% [4–6], 12–18% [4–6], 7–16% [4, 6], 11–18% [4–6], 3–6% [4–6], 7–9% [4–6], 9% [4], and 2% [4], respectively, and those frequencies were comparable to present study. There are a limited number of reports analyzing prognosis according to initial recurrence sites. A previous study reported that poor PRS has been observed in patients with lung [15], brain [16], bone [5, 6, 16, 17], and liver recurrence [6, 18]. However, since these studies were based on small sample size, there is no consensus on the association between PRS and recurrence site. In the present study of 727 recurrent patients, the largest study using a multicenter database revealed that HRS or multiple LRS recurrences were associated with a significantly poor PRS. In contrast, PRS was significantly better in patients with single LRS recurrence.

The specific reason why PRS differs in recurrence site is as follows: first, HRS including bone, brain, and pleural recurrence may decrease the patient's quality of life or lower performance status, making treatment after recurrence more difficult [19–23]. Second, recurrences in the liver and CNS have been reported to be less responsive to chemotherapy [22]. As shown in Table 2, a more aggressive tumor was observed in the HRS or multiple LRS recurrence groups than in the single LRS recurrence group, which may result in poor PRS.

Recently, it has been reported that local therapy prolongs PRS in patients with 3–5 or fewer oligo-recurrent foci [3, 24–26]. The present study suggests that local therapy may improve the prognosis of patients with a single LRS recurrence because cancer cells with a less aggressive nature are localized. Previously, Hishida et al. reported that oligo-distant recurrence (single site) has no difference in prognosis compared with oligo-locoregional recurrences (1–3 sites) [24]. Torok et al. reported oligo-distant recurrence (1–3 sites) had a significantly better PRS than diffuse distant recurrence (> 3 sites or pleural dissemination). Moreover, adrenalectomy for patients with isolated adrenal metastasis from NSCLC showed a favorable prognosis [27]. Further investigation is necessary to determine the efficacy of local therapy for the oligo-distant recurrence of a single LRS.

Patients with HRS or multiple LRS recurrences had poor PRS and OS, suggesting that these patients had systemic cancer at the time of surgery. These patients had difficulty in controlling cancer through local therapy such as surgery and radiation therapy alone, and combination therapy with systemic therapy, such as immune checkpoint inhibitors, tyrosine kinase inhibitors, and chemotherapy was necessary during the perioperative period. In recent years, nivolumab plus platinum-based chemotherapy has demonstrated longer event-free survival for clinical stage IB-IIIA NSCLC without epidermal growth factor receptor gene mutation (EGFR) and anaplastic lymphoma kinase (ALK) translocation in CheckMate-816. The HR for death or distant metastases was 0.53 (95% CI, 0.36–0.77) and the subgroup analysis showed that greater benefit was observed in a population with a poor prognosis [14].

There are few studies on predictors of recurrence in specific organs and even fewer studies on preoperative predictors. A previous study reported that tumor grade, metastatic lymph node ratio ≥ 30% (LNR), non-squamous cell carcinoma histology, bronchial invasion, perineural invasion, and adjuvant chemotherapy were associated with brain recurrence [7, 28, 29]. Motono et al. analyzed seven postoperative patients with pleural dissemination and reported that young age and poor differentiation are risk factors for pleural dissemination [30]. Previous studies have reported that older age [8], adenocarcinoma histology [8, 31], and higher stage [8, 32] are associated with distant recurrence. Wu et al. scored the risk of distal recurrence as smoking history, additional primary malignancy, non-anatomic resections, adenocarcinoma histology, pleural invasion, and angiolymphatic invasion and reported that intermediate- or high-risk groups had a higher frequency of distal recurrence [33]. The strength of this study is the analysis of preoperative CEA values and the PET maxSUV in all patients. Furthermore, the present study is the first to show that CEA values and PET maxSUV, along with CT tumor size and clinical nodal metastasis, are preoperative predictors of HRS and multiple LRS recurrences leading to poor PRS.

Patients with CEA ≥ 5 ng/ml, PET maxSUV ≥ 3.2, CT tumor size ≥ 2.4 cm, and clinical nodal metastasis recur in HRS or multiple LRS at 55.9% within 5 years after surgery; therefore, these patients should receive aggressive neoadjuvant/adjuvant therapy. On the other hand, neoadjuvant therapy may not be necessary in patients with any of these predictors, as vital metastasis occurs in as low as 3.5% of cases. These preoperative predictors are important for personalized neoadjuvant/adjuvant therapy in resectable clinical stage IB-III NSCLC. Further studies are necessary to compare the effectiveness of neoadjuvant therapy and adjuvant therapy for patients who are likely to experience recurrence at HRS or multiple LRS in a large-scale clinical trial.

This study demonstrated the importance of comprehensive surveillance of all recurrence sites at the time of recurrence to predict survival after recurrence. Moreover, recognition of HRS/LRS and the risk of recurrence are useful for postoperative surveillance. Patients with symptomatic recurrence have been reported to have a poorer prognosis than those with asymptomatic recurrence detected during surveillance [34]. Detection of a single LRS before developing into multiple LRSs may result in a better PRS. Although the ASCO guidelines do not recommend routine follow-up with PET-CT or head magnetic resonance imaging (MRI) (evidence quality: low; strength of recommendation: moderate) [35], intentional follow-up, including head MRI and PET-CT, is considered necessary for patients with one or more predictors of vital recurrence. Future clinical trials of personalized surveillance based on CEA level, PET maxSUV, tumor size and clinical nodal status are warranted.

This study has several limitations. First, this was a retrospective study and selection bias may have been possible. No common surveillance protocol has been established at the three institutions in this study, both postoperatively and at recurrence. Second, we did not investigate the effects of postoperative adjuvant therapy and post-recurrence therapy. Third, this study did not examine the number of recurrent foci. Further studies on the association between the number of recurrent foci and PRS are necessary. Fourth, information on EGFR mutations, ALK translocation, and programmed cell death 1- ligand 1 status was not available in this study. The relationship between these statuses and recurrence sites needs to be further verified. Fifth, this study included a small number of patients with skin (*n* = 5) and eye and tongue (*n* = 3) recurrences, and further studies based on a large number are necessary to analyze the prognosis of patients with these recurrence sites.

## Conclusion

The PRS of patients with HRS and multiple LRS metastases was significantly poorer than that of patients with a single LRS. The preoperative predictors for these vital recurrences were CEA elevation (≥ 5 ng/mL), high PET maxSUV (≥ 3.2), CT tumor size (≥ 2.4 cm), and clinical nodal metastasis, and 55.9% of patients with these four factors experienced recurrence within 5 years after surgery. Aggressive neoadjuvant/adjuvant therapy should be administered to these patients. Intentional follow-up is necessary for patients with one or more predictors of vital recurrence.

### Supplementary Information

**Additional file 1: Supplementary Table. **Patient characteristics of cohort 1 and cohort 2.

## Data Availability

The datasets used and/or analyzed during the current study are available from corresponding author on reasonable request.

## Abbreviations

ALK: Anaplastic lymphoma kinase
CEA: Carcinoembryonic antigen
CI: Confidence interval
CNS: Central nerve system
CT: Computed tomography
EGFR: Epidermal growth factor receptor gene mutation
HR: Hazard ratio
HRS: High-risk site
LRS: Low-risk site
MRI: Magnetic resonance imaging
maxSUV: Maximum standardized uptake value
NSCLC: Non-small cell lung cancer
PET: Positron emission tomography
PRS: Post-recurrence survival
QOL: Quality of life
TKI: Tyrosine kinase inhibitor
