# Supplementary material for: Preoperative predictors for recurrence sites associated with poor post-recurrence survival after surgery of non-small cell lung cancer: a multicenter study
Source: BMC Cancer. 2023 Nov 6;23:1064. doi: 10.1186/s12885-023-11582-y (PMC10626659; doi:10.1186/s12885-023-11582-y)
Supplement: Supplementary file 1 — Additional file 1: Supplementary Table. Patient characteristics of cohort 1 and cohort 2. [file 12885_2023_11582_MOESM1_ESM.docx]

Supplementary Table Patient characteristics of cohort 1 and cohort 2

|  | Cohort 1  (n=4520) | Cohort 2  (n=727) |
| --- | --- | --- |
| Age, median (IQR), y | 70 (64-76) | 72 (65-77) |
| Male, No. (%) | 2550 (56.4) | 512 (70.4) |
| Smoking history, No. (%) | 2788 (61.7) | 536 (73.7) |
| Brinkmann index, median (IQR) | 370 (0-940) | 760 (3-1120) |
| Right side, No. (%) | 2701 (59.8) | 423 (58.2) |
| CT tumor size, median (IQR), cm | 2.3 (1.6-3.2) | 3.6 (2.2-4.3) |
| Tumor location (lower lobe), No. (%) | 1736 (38.4) | 309 (42.5) |
| CEA 5ng/mL≦, No. (%) | 1040 (23.0) | 269 (37.0) |
| PET maxSUV, median (IQR) | 2.8 (1.2-7.2) | 7.8 (4.2-12.4) |
| Clinical stage II ≦, No. (%) | 789 (17.5) | 312 (42.9) |
| Surgical procedure, No. (%)  Wedge  Segmentectomy  Lobectomy  Pneumonectomy | 550 (12.2)  830 (18.4)  3115 (68.9)  25 (0.5) | 85 (11.7)  48 (6.6)  578 (79.5)  16 (2.2) |
| Non-adenocarcinoma, No. (%) | 927 (20.5) | 248 (34.1) |
| Lymphatic invasion +, No. (%) | 984 (21.8) | 376 (51.7) |
| Blood vessel invasion +, No. (%) | 1458 (32.3) | 514 (70.7) |
| Pleural invasion +, No. (%) | 976 (21.6) | 373 (51.3) |
| Nodal metastasis +, No. (%) | 663 (14.7) | 342 (47.0) |
| Pathological stage II ≦, No. (%) | 1076 (23.8) | 459 (63.1) |
| Recurrence sites, No. (%) |  |  |
| Lung, No. (%) |  | 309 (42.5) |
| Intrathoracic lymph node, No. (%) |  | 225 (30.9) |
| Pleura, No. (%) |  | 112 (15.4) |
| Bone, No. (%) |  | 110 (15.1) |
| Central nerve system, No. (%) |  | 86 (11.8) |
| Adrenal gland, No. (%) |  | 25 (3.4) |
| Abdominal organ, No. (%) |  | 60 (8.3) |
| Cervical and axillary lymph nodes, No. (%) |  | 38 (5.2) |
| Chest wall, No. (%) |  | 13 (1.8) |
| Skin, No. (%) |  | 5 (0.7) |
| Eye and tongue, No. (%) |  | 3 (0.4) |

CEA, carcinoembryonic antigen; CT, computed tomography; IQR, interquartile range; maxSUV, maximum standardized uptake value; PET, positron emission tomography.
